# Supplementary material for: Trogocytic molting of T cell microvilli upregulates T cell receptor surface expression and promotes clonal expansion
Source: Nat Commun. 2023 May 24;14:2980. doi: 10.1038/s41467-023-38707-y (PMC10205730; doi:10.1038/s41467-023-38707-y)
Supplement: Supplementary file 1 — supplementary information [file 41467_2023_38707_MOESM1_ESM.pdf]

SUPPLEMENTARY INFORMATION FOR

Trogocytic molting of T cell microvilli upregulates T cell receptor surface expression and promotes clonal expansion

Park, et al.

Jeong-Su Park, Jun-Hyeong Kim, Won-Chang Soh, Na-Young Kim, Kyung-Sik Lee, Chang-Hyun Kim, Ik-Joo Chung, Sunjae Lee, Hye-Ran Kim, and Chang-Duk Jun

Supplementary Figures 1-16
Captions for Supplementary Figures 1-16
Supplementary Table 1

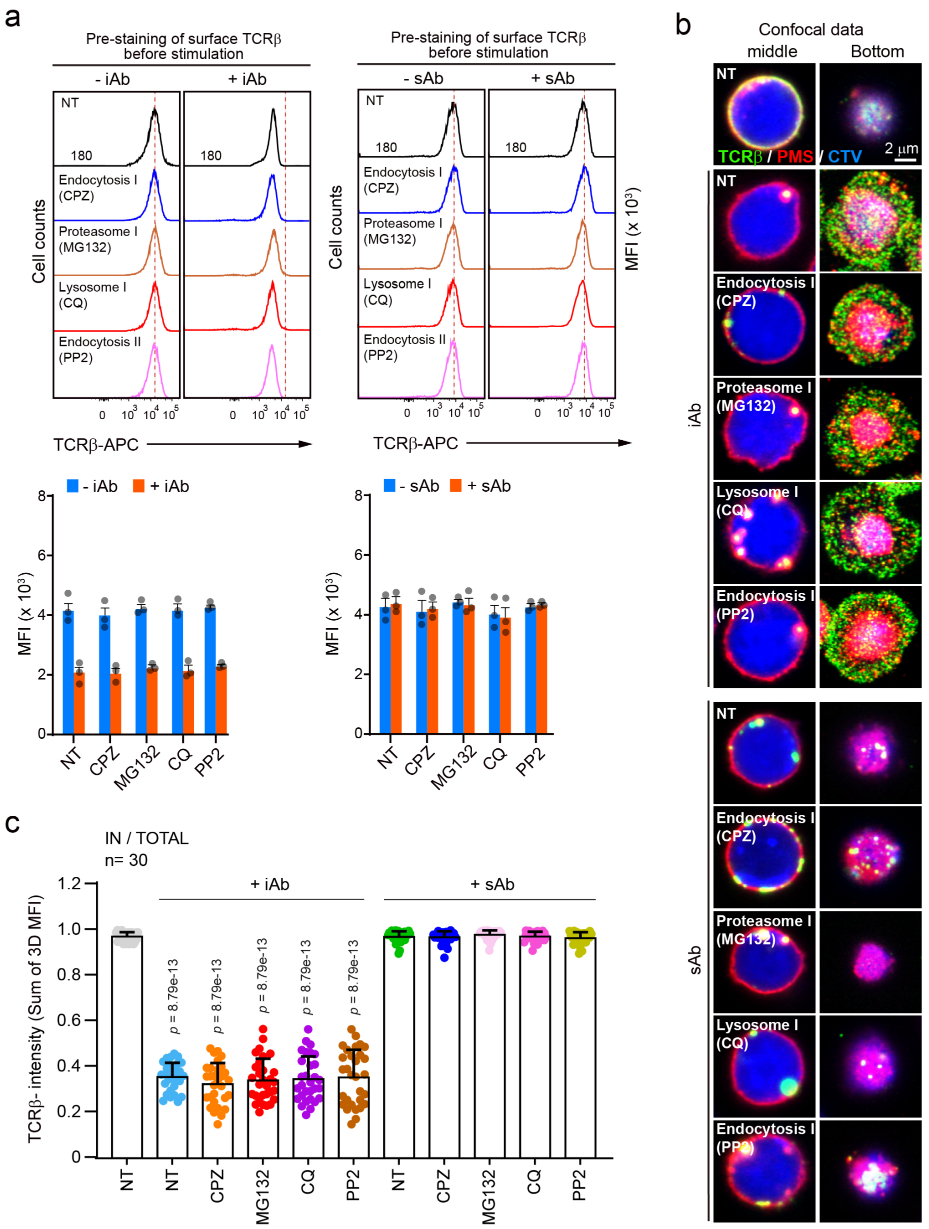

Supplementary Fig. 1. Surface TCRs downregulation is not due to internalization but is dependent on extracellular release. (a and b) Naive $CD3^+$ T cells pre-stained with anti-TCRβ-APC were treated with various inhibitors, including chlorpromazine (CPZ, 20 μM), MG132 (20 μM), chloroquine (CQ, 20 μM), or PP2 (10 μM). Cells were then stimulated with iAb or sAb for 3 h (linked experiment with Fig. 1b). The MFI of TCRβ-APC was measured by flow cytometry Results are representative of three independent experiments ± SEM. (b and c) PMS-Orange/CTV-labeled/TCRβ-stained (Alexa488) $CD3^+$ T cells were activated as in (a), and distribution of TCRβ was observed by AiryScan confocal microscopy (b). TCRζ intensity (sum of 3D MFI) was presented as inside intensity divided by total intensity (c). Results are representative of three independent experiments ± SEM. Statistics was performed using one-way ANOVA with Post hoc Tukey's multiple comparisons test (a-c). Source data are provided as a Source Data file.

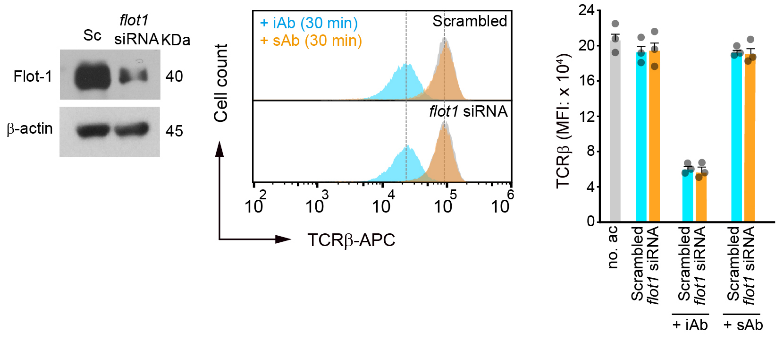

Supplementary Fig. 2. Knockdown of *flot1* little effect on TCR shedding induced by iAb. Naïve $CD3^{+}$ T cells were transfected with scrambled or siRNA targeting *flot1*. At 24 h post transfection, knock-down efficiency was confirmed by western blot. Results are representative of three independent experiments ± SEM. Cells were pre-stained with anti-TCRβ-APC and stimulated with iAb or sAb for 3 h. The loss of surface TCRs was determined by flow cytometry. Statistics was performed using unpaired two-tailed *t*-test. Source data are provided as a Source Data file.

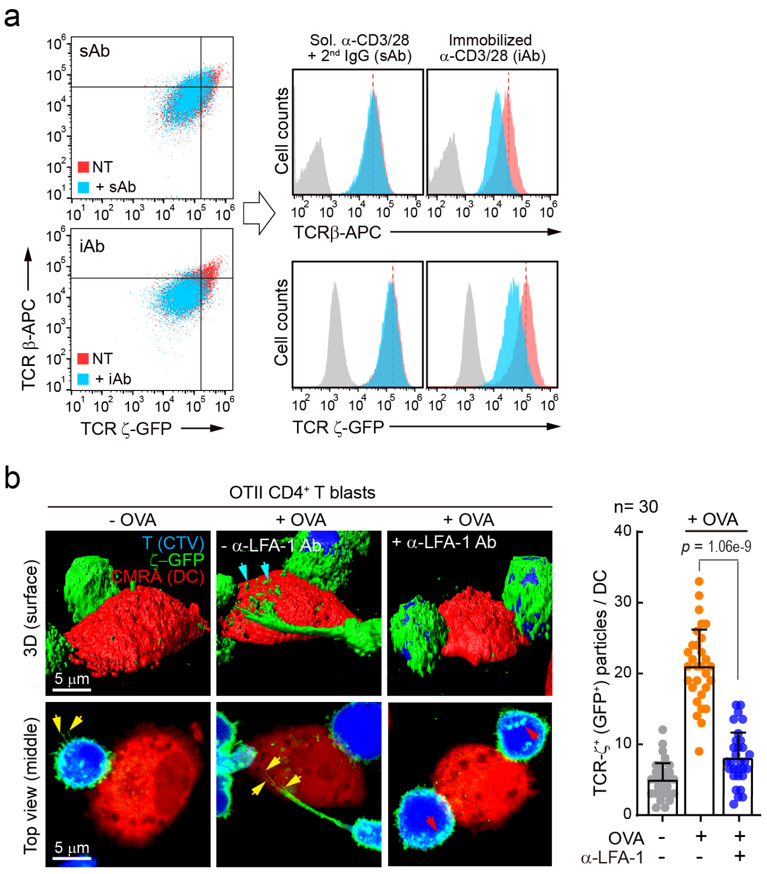

Supplementary Fig. 3. Activation of TCR ζ_GFP^{+} T blasts by iAb or pMHC-DC releases TCR ζ_GFP^{+} particles. (a) OTII CD4^{+} T cells expressing TCRζ_GFP were stained with anti-TCRβ-APC and stimulated with iAb or sAb for 3 h. The loss of surface TCRs was determined by flow cytometry. Results are representative of three independent experiments. (b) OTII CD4^{+} T cells expressing TCR ζ_GFP were stained with Cell Trace Violet (CTV) and incubated with 1 µg/mL pOVA$_{323\text{–}339}$-pulsed DCs (CMRA-Orange) in the absence or presence of anti-LFA-1 blocking antibody (10 µg/mL). Cyan Arrowheads indicate TCR ζ_GFP clusters separated from OTII CD4^{+} T cells on DCs in the presence of antigen. Yellow arrowheads represent elongated microvilli on contact site with dendritic cells. Red arrowheads indicate internalized TCR ζ_GFP in the presence of anti-LFA-1 antibodies. TCR ζ_GFP^{+} particles per single DC were quantitated using Imaris. Data represent the mean ± SD of three independent experiments (n =30). Statistics was performed using unpaired two-tailed *t*-test. Source data are provided as a Source Data file.

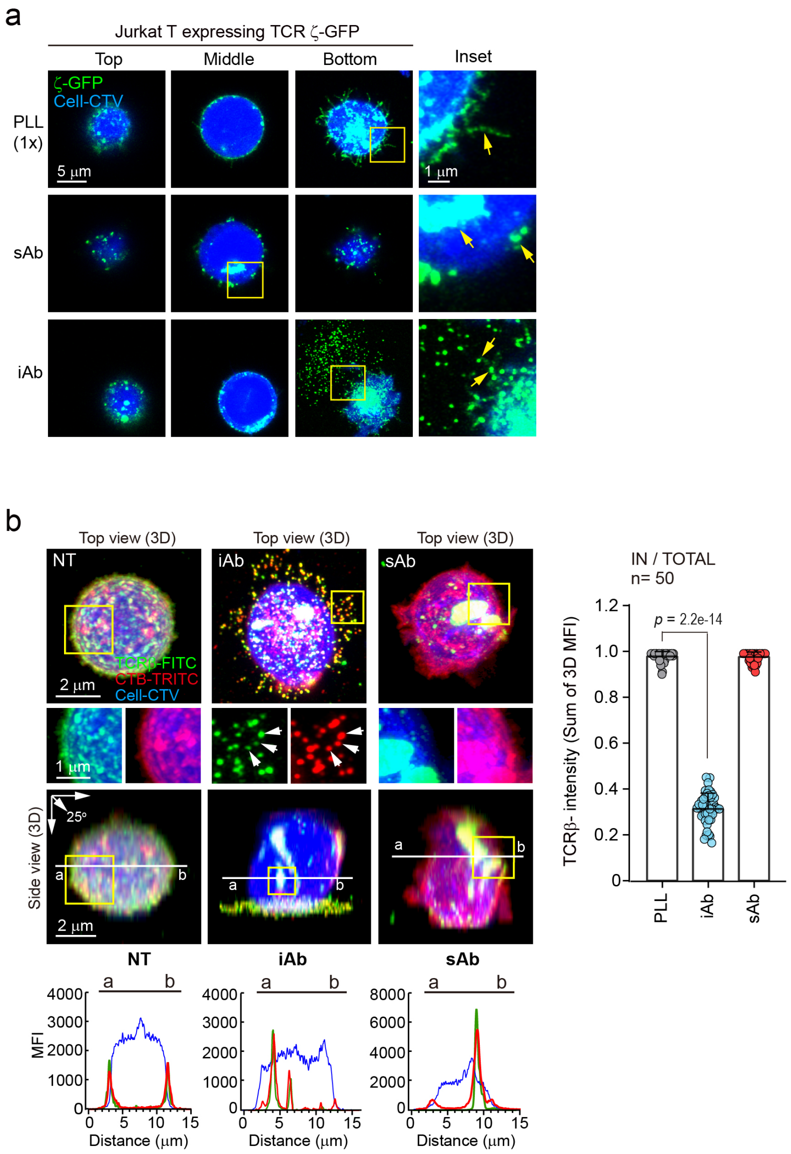

Supplementary Fig. 4. TCR ζ^+ and CTB$^+$ fluorescent signals are internalized by sAb from, but released from human and mouse T cells in iAbs. (a) Jurkat T cells expressing TCR ζ_GFP were stimulated with iAb or sAb and observed by AiryScan confocal microscopy. Yellow arrowheads indicate the TCR ζ_GFP localized at microvilli on PLL, internalized in cells in sAb condition or in released particles in iAb-stimulated cells. These results were independently repeated three times from more than 20 randomly selected cells (PLL, + sAb, + iAb respectively), and similar results were consistently obtained in all replicates (b) Localization of TCRβ (FITC) and CTB (TRITC) signals in released particles or internalized clusters stimulated with iAb or sAb for 3 h. TCR ζ_GFP intensity (sum of 3D MFI) was presented as inside intensity divided by total intensity. Data represent the mean ± SD of three independent experiments (n =50). Statistics was performed using one-way ANOVA with Post hoc Tukey's multiple comparisons test. Source data are provided as a Source Data file.

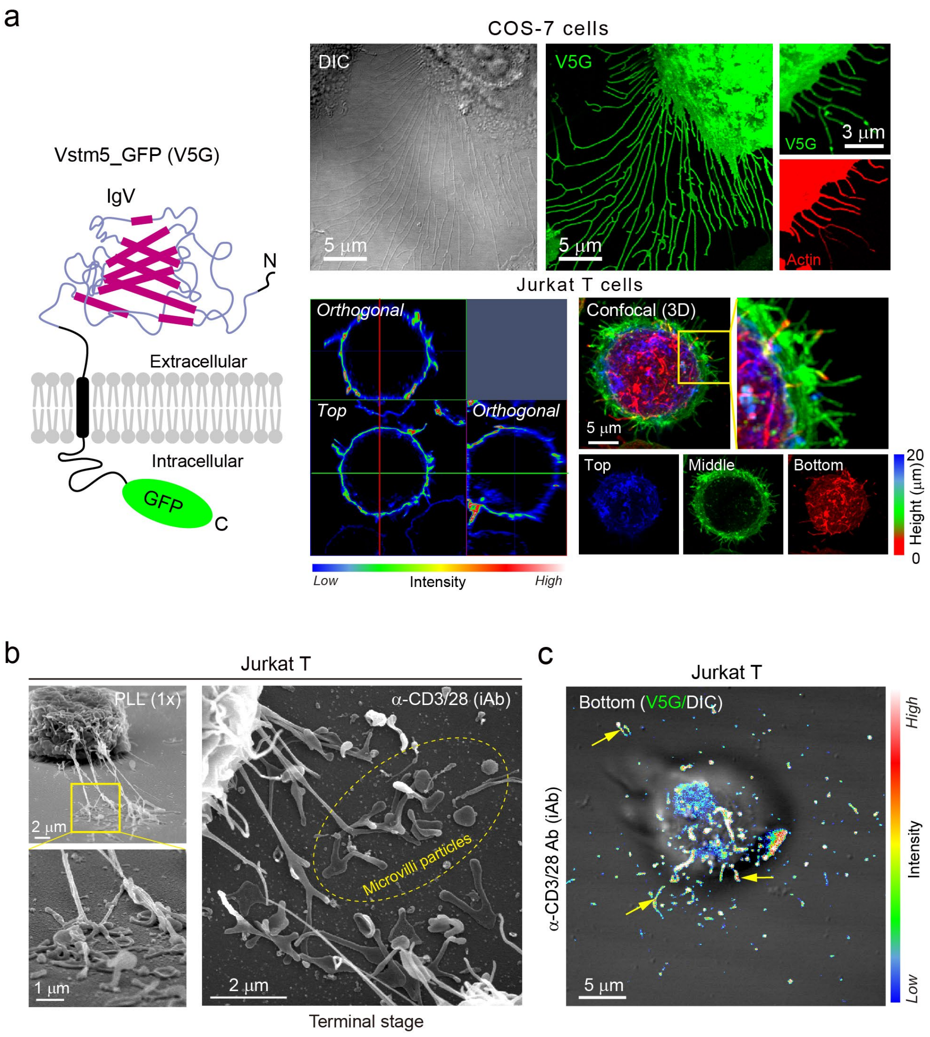

Supplementary Fig. 5. V5G is located at the finger-like membrane-protrusive region: microvilli. (a) Schematic representation of Vstm5 fused with GFP (V5G) structure on the cell membrane and subcellular localization of V5G in COS-7 or Jurkat T cells. V5G-expressing COS-7 cells were fixed and stained with phalloidin-TRITC (top). V5G in Jurkat T cells was represented as pseudo-color coding according to the fluorescence intensity (bottom). (b) SEM evidence of TIS release from Jurkat T cells. Jurkat T cells were incubated on PLL or stimulated with iAb for 60 min (terminal stage). (c) $V5G^{+}$ Jurkat cells were stimulated with iAb for 3 h and observed by confocal microscopy. V5G in microvilli was represented with pseudo-color coding according to the fluorescence intensity. Yellow arrowheads indicate the separated large TISs. These experiments were independently repeated three times from more than 20 randomly selected cells (COS-7 and Jurkat T cells), and similar results were consistently obtained in all replicates (a-c).

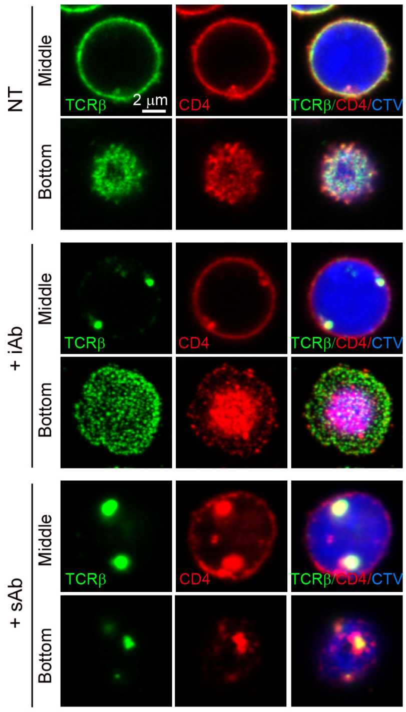

Supplementary Fig. 6. Not all CD4 and CD8 molecules are likely co-localized with TCRβ in microvilli. CTV-labeled/ anti-CD4-Alexa 549/ anti-TCRβ-Alexa 488-stained naïve $CD4^+$ T cells were stimulated with iAb or sAb for 3 h and distribution of TCRβ and CD4 was observed by AiryScan confocal microscopy. These experiments were independently repeated three times from more than 20 randomly selected cells (NT, + sAb, + iAb respectively), and similar results were consistently obtained in all replicates.

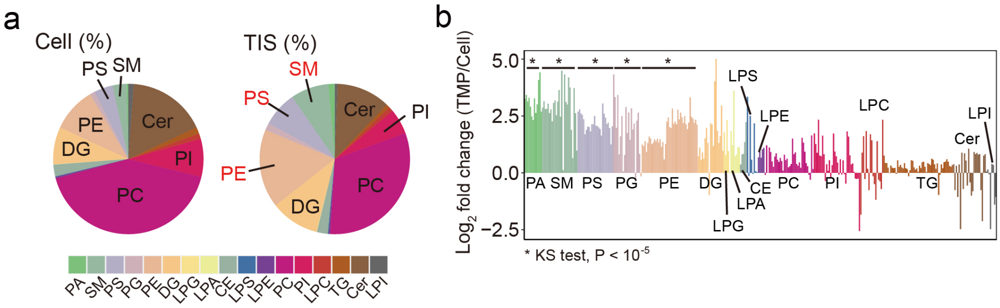

Supplementary Fig. 7. Lipid compositions enriched in TISs and total cell extracts. (a and b) Pie chart showing the global lipidomes of total cell extracts and TMPs. Lipids that were significantly enriched in TMPs compared with T cells are denoted by an asterisk (*). Source data are provided as a Source Data file.

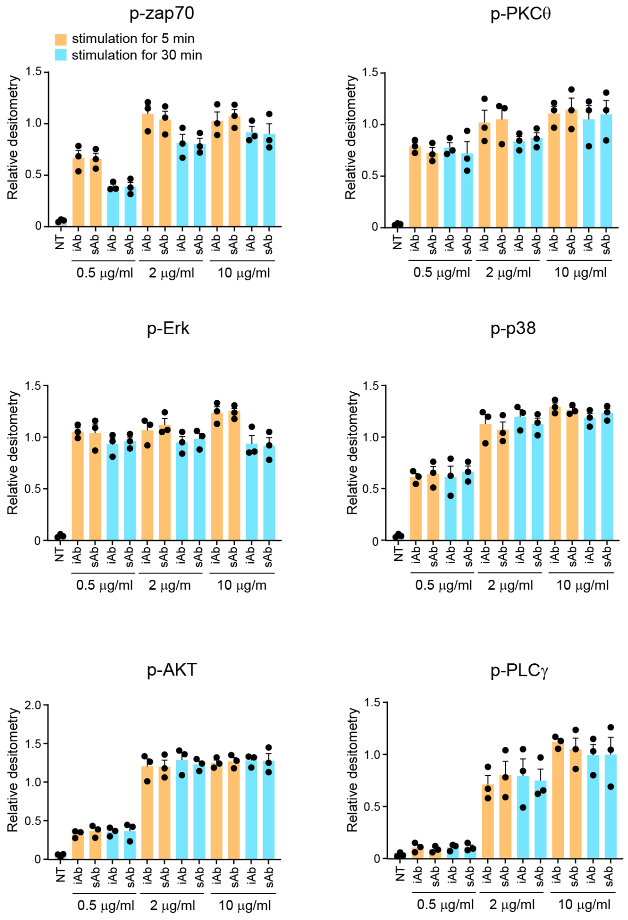

Supplementary Fig. 8. Densitometric analysis of western blot. Relative band intensities of western blot data used in Fig. 4a were statistically analyzed using image J software and GraphPad (n = 3 individual experiments). Results are representative of three independent experiments ± SEM. Statistics was performed using one-way ANOVA with Post hoc Tukey's multiple comparisons test. Source data are provided as a Source Data file.

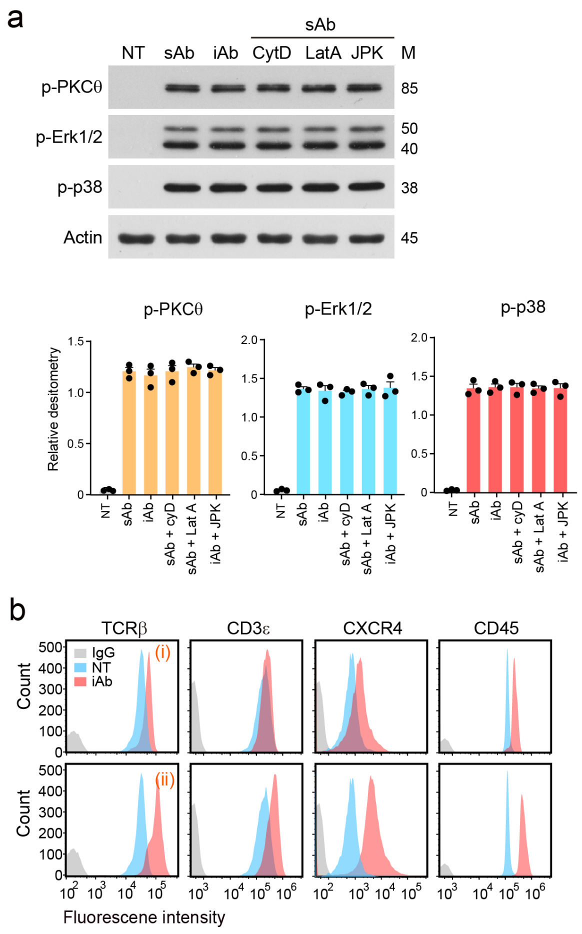

Supplementary Fig. 9. Trogocytic molting of T-cell microvilli increases cell size and surface protein expression. (a) Naive CD3^{+} T cells were stimulated with anti-CD3/CD28 (10 µg/mL, 2 µg/mL each) iAb or sAb for 3 h in the presence or absence of actin-modulating drugs, and the TCR distal signaling pathway was analyzed by western blotting. Results are representative of three independent experiments ± SEM. Statistics was performed using unpaired two-tailed *t*-test. The graph of the densitometric analysis was statistically analyzed using image J software and GraphPad (n = 3 individual experiments). The samples derive from the same experiment and that gels/blots were processed in parallel. (b) Cells from Fig. 4c gating (i) and (ii) were analyzed for levels of surface proteins potentially enriched or excluded in microvilli. Source data are provided as a Source Data file.

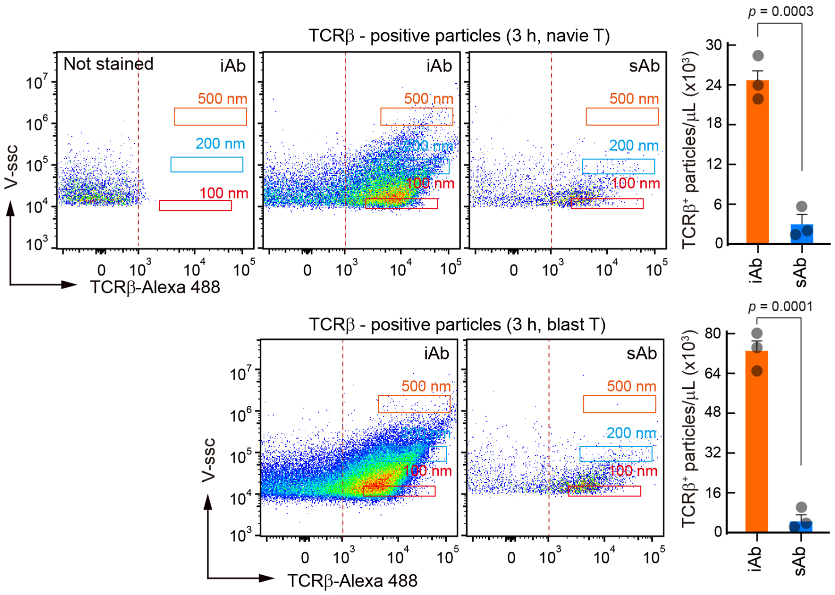

Supplementary Fig. 10. Effector T cells release more TCR$^+$TISs than naive T cells. Naive and effector CD3$^+$ T cells were pre-stained with anti-TCRβ-Alexa488 and stimulated with iAb or sAb for 3 h. The number of TCRβ$^+$ particles released were quantitated by CytoFLEX. Results are representative of three independent experiments ± SEM. *p*-value was represented in the figure *vs.* iAb. Statistics was performed using unpaired two-tailed *t*-test. Source data are provided as a Source Data file.

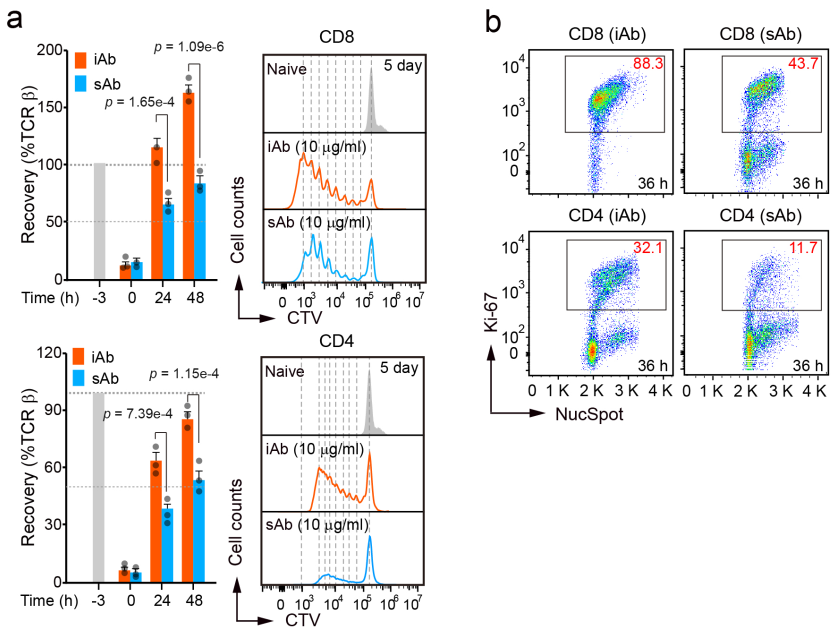

Supplementary Fig. 11. Trogocytic molting of microvilli enhances TCR recovery and T-cell proliferation in CD4$^+$ and CD8$^+$ T cells. (a and b) CTV-labeled naive CD4$^+$ or CD8$^+$ T cells were stimulated with anti-CD3/CD28 iAb or sAb for 3 h, washed, and further incubated for the indicated time periods. TCR recovery on the cell surface and cell division (a) and the proliferating populations were determined by Ki-67/NucSpot-double-positive staining at 36 h after stimulation (b). Results are representative of three independent experiments ± SEM (a and b). *p*-value was represented in the figure *vs.* iAb. Statistics was performed using one-way ANOVA with Post hoc Tukey's multiple comparisons test (a and b). Source data are provided as a Source Data file.

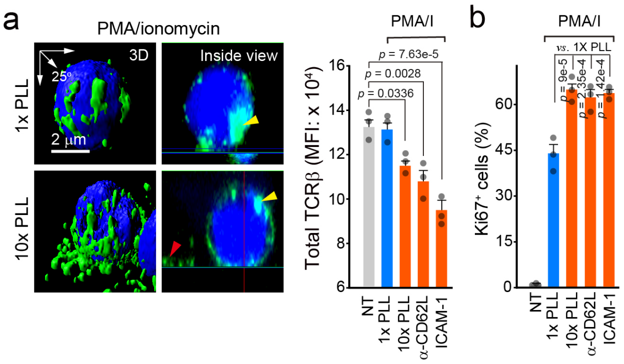

Supplementary Fig. 12. Microvilli release enhances T-cell proliferation by PMA/ionomycin. (a and b) CTV-labeled naive $CD3^{+}$ T cells were stained with anti-TCRβ-FITC) and stimulated with PMA/ionomycin (200 nM/1 μM) in the presence of indicated conditions for 3 h (continued from Fig. 5h). Distribution of $TCR\beta^{+}$ signals (GFP) and the MFI of TCRβ on the T-cell surface were analyzed (a). Data represent the mean ± SEM of three independent experiments. *p*-value was represented in the figure *vs.* NT. The proliferating cells were determined at 48 h after stimulation (b). Internalized (yellow) and separated (red) TCRβ signals were indicated. Data represent the mean ± SEM of three independent experiments. Statistics was performed using one-way ANOVA with Post hoc Tukey's multiple comparisons test. Source data are provided as a Source Data file.

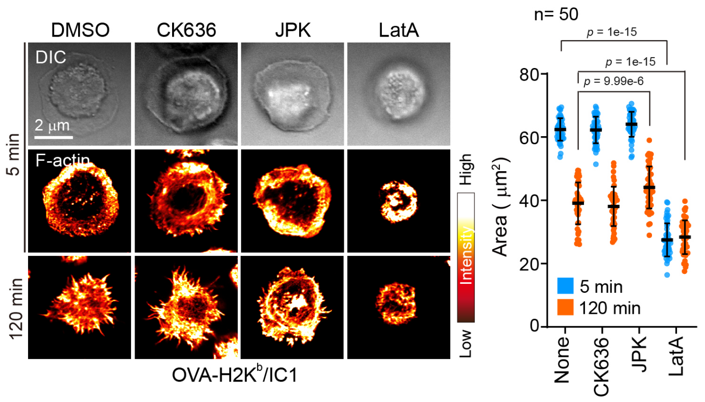

Supplementary Fig. 13. LatA and CytD significantly reduced T-cell spreading on the OVA/H-2K^b/ICAM-1. Naive CD8$^+$ T cells from *OTI* mouse were stimulated on a lipid bilayer presenting pOVA$_{257–265}$/H-2K^b/ICAM-1 in the presence of CK636 (100 μM), JPK (100 nM), or Lat A (237 nM) for 1 h, fixed, and stained with phalloidin-TRITC. Cell spreading was observed by confocal microscopy and the area (μm^2) was measured using the ImageJ software. Results are representative of three independent experiments ± SD. Statistics was performed using one-way ANOVA with Post hoc Tukey's multiple comparisons test. Source data are provided as a Source Data file.

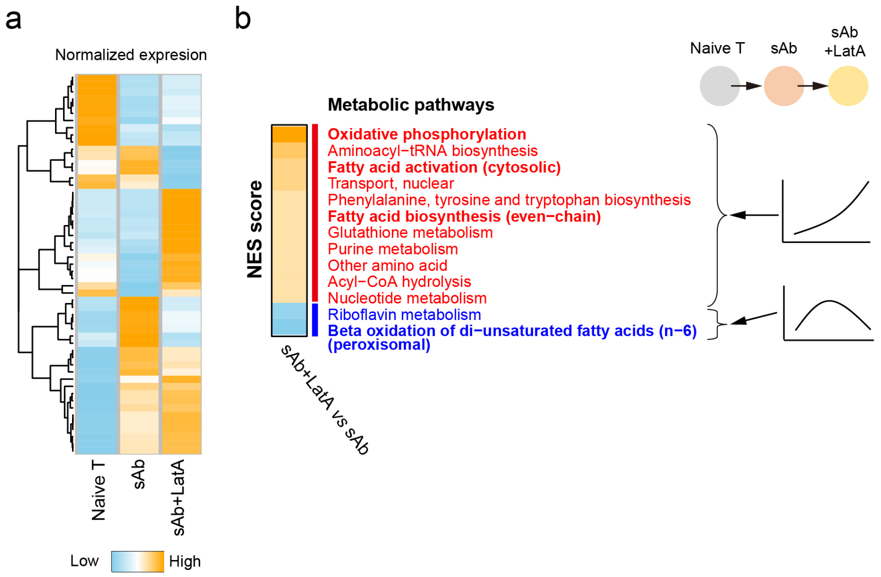

Supplementary Fig. 14. Enrichment tests of metabolic pathways of T cells. (a) Normalized enrichment scores (NES) of metabolic pathways (R fgsea package) were calculated from the fold changes between sAb+LatA-activated T cells and sAb-activated T cells (at 3 h). (b) Increased expression of metabolic pathways, including OXPHOS, FAS, and nucleotide metabolism, and decreased expressions of metabolic pathways, including FAO, were observed. Pathways of the heatmap were selected based on enrichment test p values (<0.05).

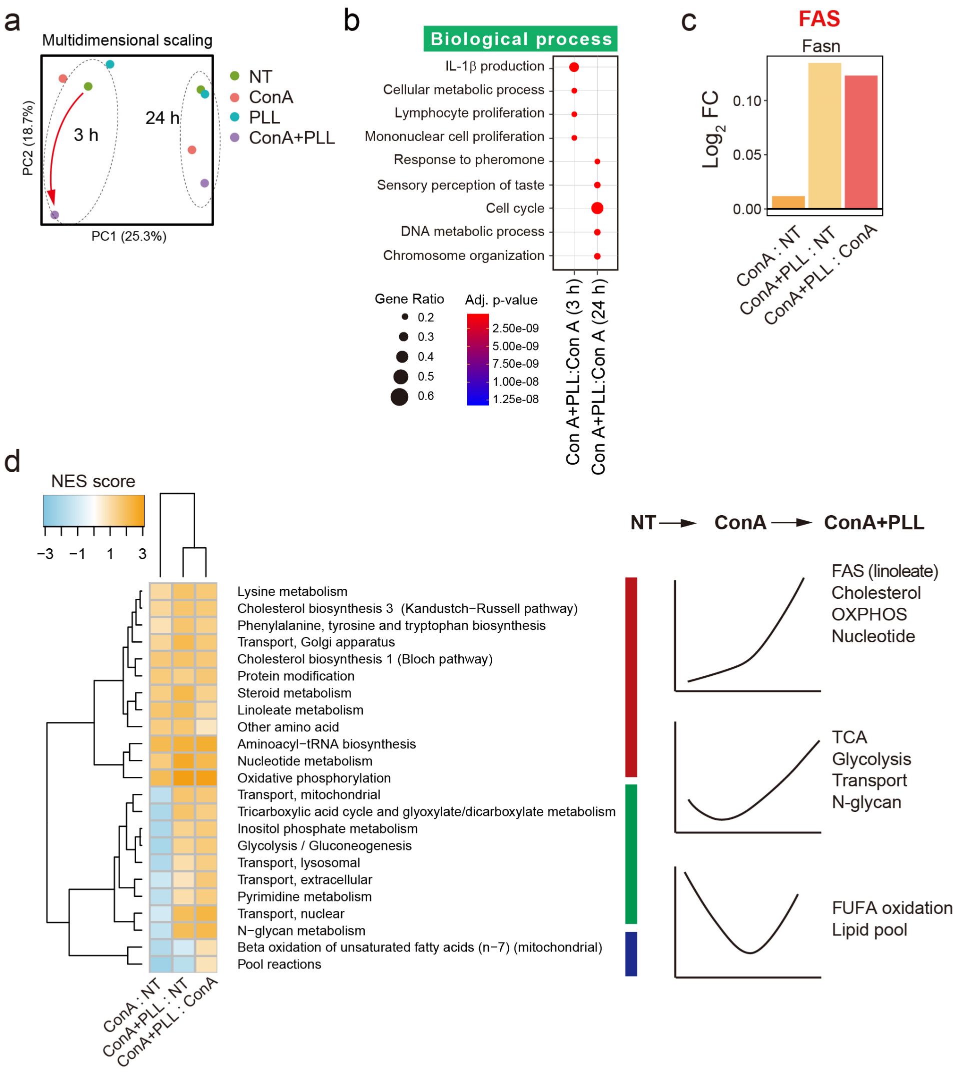

Supplementary Fig. 15. Transcriptome analysis of naive T cells and ConA- and ConA plus PLL-activated naive T cells. (a) Principal component analysis (PCA) of transcriptome. Trajectory was changed from naive T cell to PLL-, ConA-, and ConA+PLL-activated (3 and 24 h) T cells. The changes were time-dependent, observing different clusters by time. (b) Enriched biological pathways (Gene Ontology) of differentially expressed genes. (c) Bar graphs show the fold changes (log2) of representative genes in FAS pathways. (d) Normalized enrichment scores (NES) of metabolic pathways (R fgsea package) were calculated from the fold changes among ConA *vs*. naive T, ConA+PLL *vs*. naive T, and ConA+PLL *vs*. ConA.

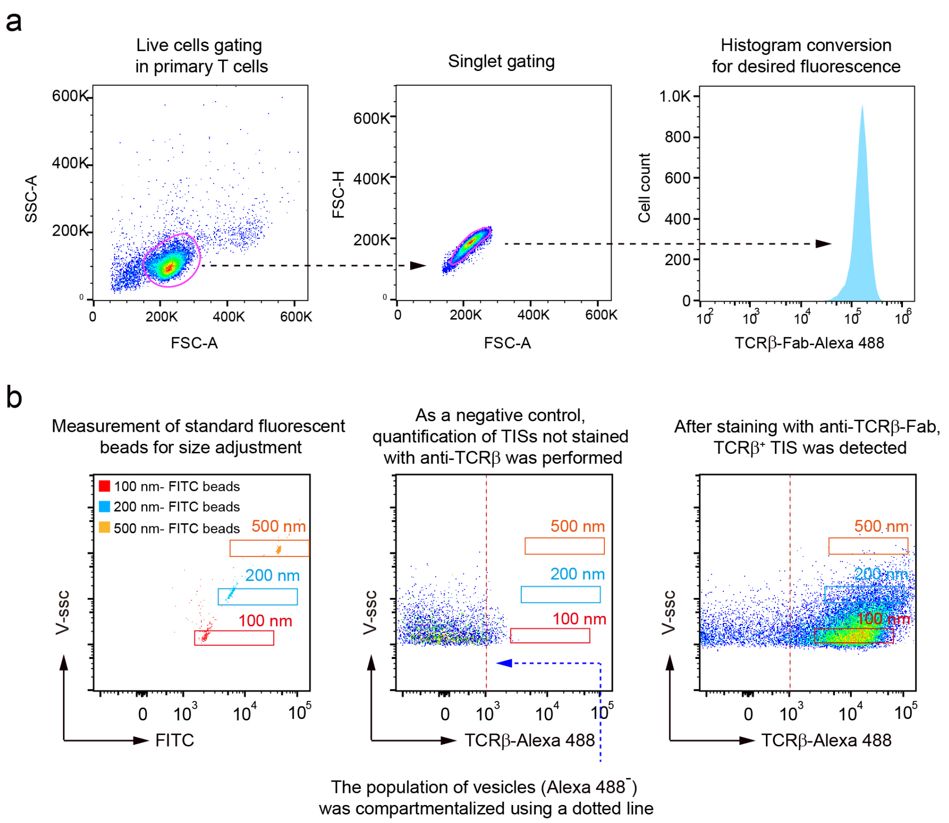

Supplementary Fig. 16. Gating strategies for flow cytometry analysis. (a) Measurement of surface expression of target proteins. After isolating T cells from lymph nodes and spleen in C57BL/6 mouse, lymphocytes were gated based on SSC-A versus FSC-A and singlets were selected from the FSC-A versus FSC-H dot plot. The surface expression of the indicated proteins was measured by gating only live cells. (b) Quantification of TISs. Isolated TISs was quantified by size- and fluorescent-based methods as described in Quantitation of TISs by flow cytometry in Methods section. All FACS analyses were represented as histograms, dot plot, bar graphs using specified gating strategy.

Supplementary Table 1. Antibody information

Name	Clone	Vendor	Catalog#	Dilution
Anti-TCRβ	H57-597 (HB218)	Bio-X-Cell	BE0102	1:200
CD3ε	145-2C11	Biolegend	100305	1:200
CD62L	MEL-14	Biolegend	104412	1:200
CD25	3C7	Biolegend	101904	1:200
CD45	I3/2.3	Biolegend	147708	1:200
CD11c	N418	Biolegend	117308	1:200
CD69	H1.2F3	Biolegend	104514	1: 200
CD4	RM4-5	Biolegend	100510	1: 200
CD4	GK1.5	Biolegend	100446	1: 200
GFP	unspecified	Abcam	ab290	1:1000
CD3ζ	6B10.2	Santa Cruz Biotechnology	sc-1239	1:200
Flot1	C-2	Santa Cruz Biotechnology	sc-74566	1:200
β-Actin	unspecified	Cell Signaling Technology	4967L	1:2000
phospho-Zap70	unspecified	Cell Signaling Technology	2701S	1:2000
phospho-PKCδ/θ	unspecified	Cell Signaling Technology	9376S	1:2000
Phospho-p44/42 MAPK (Erk1/2)	20G11	Cell Signaling Technology	4376S	1:2000
Phospho-P38	unspecified	Cell Signaling Technology	9215S	1:2000
Phospho-Akt	unspecified	Cell Signaling Technology	4058S	1:2000
anti-rabbit IgG-HRP	unspecified	Cell Signaling Technology	7074S	1:2000
anti-mouse IgG-HRP	unspecified	Cell Signaling Technology	7076S	1:2000
anti-mouse CD3	145-2C11; CRL-1975	BioXcell	BE0001-1	2 or 10 µg/ml
anti-mouse CD28	PV1; HB-12352,	BioXcell	BE0015-5	2 µg/ml
anti-human CD3	OKT3; CRL-8001	BioXcell	BE0001-2	10 µg/ml
anti-human CD28	37407	R&D Systems	BE0248	2 µg/ml
anti-Armenian Hamster IgG (H+L) Secondary Antibody	unspecified	Invitrogen	31115	2 µg/ml
Goat anti-Mouse IgG (H+L), Superclonal™ Recombinant Secondary Antibody	unspecified	Invitrogen	A28174	2 µg/ml
anti-CD62L	MEL-14	BioXcell	BE0021	10 µg/ml
Recombinant Mouse	unspecified	R&D Systems	10304-IC	10 µg/ml

ICAM-1/CD54 His-tag				
Recombinant Mouse VCAM-1/CD106 Fc	unspecified	R&D Systems	643-VM-200	10 µg/ml
Fibronectin	unspecified	Sigma Aldrich	F2006	10 µg/ml
