# Supplementary material for: Trogocytic molting of T cell microvilli upregulates T cell receptor surface expression and promotes clonal expansion
Source: Nat Commun. 2023 May 24;14:2980. doi: 10.1038/s41467-023-38707-y (PMC10205730; doi:10.1038/s41467-023-38707-y)
Supplement: Supplementary file 3 — Reporting Summary [file 41467_2023_38707_MOESM3_ESM.pdf]

nature portfolio

Corresponding author(s): Dr. Chang-Duk Jun

Last updated by author(s): Mar 30, 2023

Reporting Summary

Nature Portfolio wishes to improve the reproducibility of the work that we publish. This form provides structure for consistency and transparency in reporting. For further information on Nature Portfolio policies, see our Editorial Policies and the Editorial Policy Checklist.

Statistics

For all statistical analyses, confirm that the following items are present in the figure legend, table legend, main text, or Methods section.

n/a	Confirmed	
☐	☒	The exact sample size (*n*) for each experimental group/condition, given as a discrete number and unit of measurement
☐	☒	A statement on whether measurements were taken from distinct samples or whether the same sample was measured repeatedly
☐	☒	The statistical test(s) used AND whether they are one- or two-sided<br>*Only common tests should be described solely by name; describe more complex techniques in the Methods section.*
☐	☒	A description of all covariates tested
☐	☒	A description of any assumptions or corrections, such as tests of normality and adjustment for multiple comparisons
☐	☒	A full description of the statistical parameters including central tendency (e.g. means) or other basic estimates (e.g. regression coefficient) AND variation (e.g. standard deviation) or associated estimates of uncertainty (e.g. confidence intervals)
☐	☒	For null hypothesis testing, the test statistic (e.g. *F*, *t*, *r*) with confidence intervals, effect sizes, degrees of freedom and *P* value noted<br>*Give P values as exact values whenever suitable.*
☒	☐	For Bayesian analysis, information on the choice of priors and Markov chain Monte Carlo settings
☐	☒	For hierarchical and complex designs, identification of the appropriate level for tests and full reporting of outcomes
☒	☐	Estimates of effect sizes (e.g. Cohen's *d*, Pearson's *r*), indicating how they were calculated

Our web collection on statistics for biologists contains articles on many of the points above.

Software and code

Policy information about availability of computer code

Data collection	Flowjo v10 (Tree star Inc.,OR,USA), GraphPad Prism 8 (CA, USA), Affymetrix® GeneChip Command Console® Software, Zen 3.0 (black edition, Carl Zeiss, Germany), Image J (https://imagej.nih.gov/), FV31S-SW (Olympus, Japan), Cytoscape V3.6.1 (http://cytoscape.org/)
Data analysis	* Flow cytometer data (.fcs file) were analyzed usingFlowjo v10 (Tree star Inc.,OR,USA).<br>* All statistical data was analyzed using GraphPad Prism 8 (CA, USA).<br>* Microarray data generation and anlysis was performed using Affymetrix® GeneChip Command Console® Software (AGCC).<br>* The analysis of Airy scan (confocal) images was performed using Zen 3.0 (black edition, Carl Zeiss, Germany) for calculation and analysis.<br>* The number of T-cell microvilli in SEM images, was quantified using the FiloQuant plugin provided by Image J.<br>* The acquisition and analysis of confocal images were performed using FV31S-SW (Olympus, Japan).<br>* The proteomics analysis in Figure 9 was performed using Cytoscape V3.6.1 (http://cytoscape.org/) with the ClueGO V2.5.7 plugin

For manuscripts utilizing custom algorithms or software that are central to the research but not yet described in published literature, software must be made available to editors and reviewers. We strongly encourage code deposition in a community repository (e.g. GitHub). See the Nature Portfolio guidelines for submitting code & software for further information.

Data

Policy information about availability of data

All manuscripts must include a data availability statement. This statement should provide the following information, where applicable:

- Accession codes, unique identifiers, or web links for publicly available datasets
- A description of any restrictions on data availability
- For clinical datasets or third party data, please ensure that the statement adheres to our policy

The LC-MS/MS data generated in this study have been deposited in the PRIDE under accession code PXD041442 and microarray data have been deposited in the GEO under accession code GSE228653 and GSE228654. The other data are available in the article, Supplementary Information, or Source Data file. Source data are provided with this paper.

Human research participants

Policy information about studies involving human research participants and Sex and Gender in Research.

Reporting on sex and gender	N/A
Population characteristics	N/A
Recruitment	N/A
Ethics oversight	N/A

Note that full information on the approval of the study protocol must also be provided in the manuscript.

Field-specific reporting

Please select the one below that is the best fit for your research. If you are not sure, read the appropriate sections before making your selection.

☒ Life sciences ☐ Behavioural & social sciences ☐ Ecological, evolutionary & environmental sciences

For a reference copy of the document with all sections, see nature.com/documents/nr-reporting-summary-flat.pdf

Life sciences study design

All studies must disclose on these points even when the disclosure is negative.

Sample size	Sample sizes were based on those used in previous and preliminary studies from our lab.
Data exclusions	No exclusion were made.
Replication	Experimental replicates were indicated in the figure legends, and each experiment was repeated at least three times.
Randomization	All experiments were conducted using primary T cells derived from randomly assigned animals or tissues derived from randomly assigned animals.
Blinding	Data reported for some experiments are not subjective but rather base on quantitative flow cytometry or quantification analysis software.

Reporting for specific materials, systems and methods

We require information from authors about some types of materials, experimental systems and methods used in many studies. Here, indicate whether each material, system or method listed is relevant to your study. If you are not sure if a list item applies to your research, read the appropriate section before selecting a response.

Materials & experimental systems

n/a	Involved in the study
	[x] Antibodies
	[x] Eukaryotic cell lines
[x]	Palaeontology and archaeology
	[x] Animals and other organisms
[x]	Clinical data
[x]	Dual use research of concern

Methods

n/a	Involved in the study
[x]	ChIP-seq
	[x] Flow cytometry
[x]	MRI-based neuroimaging

Antibodies

Antibodies used

<Antibodies for Flow cytometry>
* Anti-TCRβ (used for Fab materials, clone: H57-597 (HB218), unconjugate, manufacturer: Bio-X-Cell, cat# BE0102, dilution 1: 200)
* CD3ε (conjugated-FITC, clone: 145-2C11, manufacturer: Biolegend, cat# 100305; dilution 1: 200)
* CD62L (conjugated-APC, clone: MEL-14, manufacturer: Biolegend, cat# 104412; dilution 1: 200)
* CD25 (conjugated-PE, clone: 3C7, manufacturer: Biolegend, cat# 101904; dilution 1: 200)
* CD45 (conjugated-APC, clone: I3/2.3, manufacturer: Biolegend, cat# 147708; dilution 1: 200)
* CD11c (conjugated-PE, clone: N418, manufacturer: Biolegend, cat# 117308; dilution 1: 200)
* CD69 (conjugated-APC, clone: H1.2F3, manufacturer: Biolegend, cat# 104514; dilution 1: 200)
* CD4 (conjugated-FITC, clone: RM4-5, manufacturer: Biolegend, cat# 100510; dilution 1: 200)
* CD4 (conjugated-Alexa 594, clone: GK1.5, manufacturer: Biolegend, cat# 100446; dilution 1: 200)

<Western blot>
* GFP (unconjugated clone: unspecified, manufacturer: Abcam, cat# ab290; dilution 1:1000)
* CD3ζ (unconjugated clone: 6B10.2, manufacturer: Santa Cruz Biotechnology, cat# sc-1239; dilution 1:200)
* Flot1 (unconjugated clone: C-2, manufacturer: Santa Cruz Biotechnology, cat# sc-74566; dilution 1:200)
* β-Actin (unconjugated clone: unspecified, manufacturer: Cell Signaling Technology, cat# 4967L; dilution 1:2000)
* phospho-Zap70 (unconjugated clone: unspecified, manufacturer: Cell Signaling Technology, cat# 2701S; dilution 1:2000)
* phospho-PKCδ/θ (unconjugated clone: unspecified, manufacturer: Cell Signaling Technology, cat# 9376S; dilution 1:2000)
* Phospho-p44/42 MAPK (Erk1/2) (unconjugated clone: 20G11 manufacturer: Cell Signaling Technology, cat# 4376S; dilution 1:2000)
* Phospho-P38 (unconjugated clone: unspecified, manufacturer: Cell Signaling Technology, cat# 9215S; dilution 1:2000)
* Phospho-Akt (unconjugated clone: unspecified, manufacturer: Cell Signaling Technology, cat# 4058S; dilution 1:2000)
* anti-rabbit IgG-HRP (unconjugated clone:unspecified, manufacturer: Cell Signaling Technology, cat# 7074S; dilution 1:2000)
* anti-mouse IgG-HRP (unconjugated clone: unspecified, manufacturer: Cell Signaling Technology, cat# 7076S; dilution 1:2000)

<T-cell Activation>
* anti-mouse CD3 (unconjugated clone: 145-2C11; CRL-1975, manufacturer: American Type Culture Collection & BioXcell, cat# BE0001-1; dilution 2 or 10 μg/ml)
* anti-mouse CD28 (unconjugated, clone: PV1; HB-12352, manufacturer: American Type Culture Collection & BioXcell, cat# BE0015-5 dilution 2 μg/ml)
* anti-human CD3 (unconjugated, clone: OKT3; CRL-8001, manufacturer: American Type Culture Collection & BioXcell, cat# BE0001-2; dilution 10 μg/ml)
* anti-human CD28 (unconjugated, clone: 37407, manufacturer: R&D Systems, cat# BE0248; dilution 2 μg/ml)
* anti-Armenian Hamster IgG (H+L) Secondary Antibody (unconjugated, clone: unspecified, manufacturer: Invitrogen, cat# 31115; dilution 2 μg/ml)
* Goat anti-Mouse IgG (H+L), Superclonal™ Recombinant Secondary Antibody (unconjugated, clone: unspecified, manufacturer: Invitrogen, cat# A28174; dilution 2 μg/ml)

<Adhesion matrix>
* anti-CD62L (unconjugated clone: MEL-14, manufacturer: BioXcell, cat# BE0021; dilution 10 μg/ml)
* Recombinant Mouse ICAM-1/CD54 His-tag (conjugated-his tag, clone: mouse myeloma cell line, NS0-derived mouse ICAM-1/CD54 protein, manufacturer: R&D Systems, cat# : 10304-IC; dilution 10 μg/ml)
* Recombinant Mouse VCAM-1/CD106 Fc (unconjugated, clone: mouse myeloma cell line, NS0-derived mouse VCAM-1/CD106 protein, manufacturer: R&D Systems, cat# : 643-VM-200; dilution 10 μg/ml)
* Fibronectin (unconjugated, clone: human plasma cat#: F2006, dilution 10 μg/ml)

Validation

<<Antibodies for Flow cytometry>>
* Anti-TCRβ (Bio-X-Cell, cat# BE0102) RRID: AB_10950158
* CD3ε (Biolegend, cat# 100305) RRID: AB_312670
* CD62L (Biolegend, cat# 104412) RRID: AB_313099
* CD25 (Biolegend, cat# 101904) RRID: AB_312847
* CD45 (Biolegend, cat# 147708) RRID: AB_2563540
* CD11c (Biolegend, cat# 117308) RRID: AB_313777
* CD69 (Biolegend, cat# 104514) RRID: AB_492843
* CD4 (Biolegend, cat# 100510) RRID: AB_312713

* CD4 (Biolegend, cat# 100446) RRID: AB_2563182

<Western blot>

* Na+K+ATPase (Abcam, cat# ab7671) RRID: AB_306023
* GFP (Abacm, cat# ab290) RRID: AB_303395
* TSG101 (Abacm, cat# ab125011) RRID: AB_10974262
* Rab11 (Abacm, cat# ab3612) RRID: AB_10861613
* CD3ζ (Santa Cruz Biotechnology, Cat# sc-1239, RRID:AB_627020)
* Flot1 (Santa Cruz Biotechnology, Cat# sc-74566, RRID:AB_2106563)
* β-Actin (Cell Signaling Technology, Cat# 4967, RRID:AB_330288)
* phospho-Zap70 (Cell Signaling Technology, Cat# 2701, RRID:AB_331600)
* phospho-PKCδ/θ (Cell Signaling Technology, Cat# 9376, RRID:AB_2168834)
* phospho-p44/42 MAPK (Cell Signaling Technology, Cat# 4376, RRID:AB_331772)
* Phospho-P38 (Cell Signaling Technology, Cat# 9215, RRID:AB_331762)
* Phospho-Akt (Cell Signaling Technology, Cat# 4058, RRID:AB_331168)
* anti-rabbit IgG-HRP (Cell Signaling Technology, Cat# 7074, RRID:AB_2099233)
*anti-mouse IgG-HRP (Cell Signaling Technology, Cat# 7076, RRID:AB_330924)

<T-cell Activation>
* anti-mouse CD3 (BioXcell, cat# BE0001-1) RRID: AB_1107634
* anti-mouse CD28 (BioXcell, cat# BE0015-5) RRID: AB_1107628
* anti-human CD3 (BioXcell, cat# BE0001-2) RRID: AB_1107632
* anti-human CD28 (R&D Systems, cat# MAB342) RRID: AB_2073708
* anti-Armenian Hamster IgG (H+L) Secondary Antibody (Invitrogen, cat# 31115) RRID: AB_228247
* Goat anti-Mouse IgG (H+L), Superclonal™ Recombinant Secondary Antibody (Invitrogen, cat# A28174) RRID: AB_2536160

<Adhesion matrix>
* anti-CD62L (BioXcell, cat# BE0021) RRID: AB_1107665

Eukaryotic cell lines

Policy information about cell lines and Sex and Gender in Research

Cell line source(s)	Jurkat T cells (ATCC, TIB-152) or COS-7 cells (ATCC, CRL-1651) .
Authentication	Cell lines used in this study were authenticated by the respective sources.
Mycoplasma contamination	mycoplasma contamination was not detected both Jurkat T and COS-7 cells.
Commonly misidentified lines (See ICLAC register)	No misidentified cell line were used in this study.

Animals and other research organisms

Policy information about studies involving animals; ARRIVE guidelines recommended for reporting animal research, and Sex and Gender in Research

Laboratory animals	The experimental procedures and appropriateness of this study were approved by the Institutional Animal Care and Use Committee (IACUC) at Gwangju Institute of Science and Technology (GIST). The Laboratory Animal Resource Center (LARC), which adheres to the guidelines of the Association for Assessment and Accreditation of Laboratory Animal Care International, managed the maintenance and importation procedures of the experimental animals
Wild animals	No wild animals were used in the study.
Reporting on sex	This study was conducted using 8-week-old female C57BL/6 mice. However, this does not imply that the results are reproducible only in females, as the same results are also reproducible in males
Field-collected samples	No field collected samples were used in the study.
Ethics oversight	All experimental methods and protocols were approved by the Institutional Animal Care and Use Committee of the School of Life Sciences, Gwangju Institute of Science and Technology, and carried out in accordance with their approved guidelines (IACUC GIST-2020-107, GIST-2020-026, GIST-2017-103, and GIST-2018-053).

Note that full information on the approval of the study protocol must also be provided in the manuscript.

Flow Cytometry

Plots

Confirm that:

- [x] The axis labels state the marker and fluorochrome used (e.g. CD4-FITC).
- [x] The axis scales are clearly visible. Include numbers along axes only for bottom left plot of group (a 'group' is an analysis of identical markers).
- [x] All plots are contour plots with outliers or pseudocolor plots.
- [x] A numerical value for number of cells or percentage (with statistics) is provided.

Methodology

Sample preparation	Naïve CD3+ T cells were isolated from the lymph nodes and spleen of C57BL/6 mice. The tissue was dissociated into single-cells using a 40 µm strainer (Falcon, NY, USA). Red blood cells were removed using RBC lysis buffer (Biolegend, San Diego, CA, USA). These lymphocytes were then separated into CD3+ (CD4+ or CD8+) T cells using a cocktail of antibodies and magnetic beads provided by the MojoSort Mouse CD3 T Cell Isolation Kit (Biolegend, San Diego, CA, USA).
Instrument	FACS Canto flow cytometer (Becton Dickinson, Franklin Lakes, NJ, USA)
Software	The data obtained from FACS was converted into FCS files and analyzed using Flowjo 10
Cell population abundance	Cell population abundance within post-sort fractions is presented in Supplementary Fig. 16 The purity was determined by calculating the proportion of the desired cell fraction within the parent population of living cells. To highlight certain populations, shapes were used to indicate them in the manuscript
Gating strategy	Live lymphocytes were gated as a distinct population based on FSC/SSC properties. When confirming the expression of specific protein markers, staining was performed prior to FACS analysis and live cells were gated. Then, all live cells of having the desired fluorescence were displayed in dot plot or histogram without any further modification. The schematic diagram of the strategy is presented in Supplementary Figure 16.

- [x] Tick this box to confirm that a figure exemplifying the gating strategy is provided in the Supplementary Information.
