# Supplementary material for: Trogocytic molting of T cell microvilli upregulates T cell receptor surface expression and promotes clonal expansion
Source: Nat Commun. 2023 May 24;14:2980. doi: 10.1038/s41467-023-38707-y (PMC10205730; doi:10.1038/s41467-023-38707-y)
Supplement: Supplementary file 4 — Description of Additional Supplementary Files [file 41467_2023_38707_MOESM4_ESM.docx]

**Supplementary Movie 1. T cells do not release TCRβ^+^ fluorescence signals in the absence of antigen on DCs.** OTII CD4^+^ T cells expressing TCRζ_GFP were stained with CTV (cytosol), and the cells were incubated for 3 h with DCs (CMRA-Orange) without antigen peptide. Images were 3D-reconstituted, and the IsoSurface module of Imaris was applied. *Abbreviations*: CTV, CellTrace violet; DC, dendritic cells; OVA, ovalbumin; 3D, three-dimensional.

**Supplementary Movie 2. DCs acquire TCRβ^+^ fluorescence signals from T cells in an antigen- and adhesion-dependent manner.** OTII CD4^+^ T cells expressing TCRζ_GFP were stained with CTV (cytosol), and the cells were incubated with 1 μg/mL pOVA_323–339_-pulsed DCs (CMRA-Orange) for 3 h. Images were 3D-reconstituted, and the IsoSurface module of Imaris was applied. *Abbreviations*: CTV, CellTrace violet; DC, dendritic cells; OVA, ovalbumin; 3D, three-dimensional.

**Supplementary Movie 3. Anti-LFA-1 antibody blocks the release of TCRβ^+^ particles from activated T cells on DCs.** OTII CD4^+^ T cells expressing TCRζ_GFP were stained with CTV (cytosol), and then the cells were incubated with 1 μg/mL pOVA_323–339_-pulsed DCs (CMRA-Orange) in the presence of anti-LFA-1 blocking antibody (10 μg/mL). Images were 3D-reconstituted, and the IsoSurface module of Imaris was applied. *Abbreviations*: CTV, CellTrace violet; DC, dendritic cell; LFA, leukocyte function-associated antigen; OVA, ovalbumin.

**Supplementary Movie 4. Colocalization of TCRβ^+^ (FITC, green) and CTB^+^ (TRITC, orange) signals on the surface of unstimulated naive T cells.** *Abbreviations*: CTB, cholera toxin B subunit; V5G, Vstm5 fused with green fluorescent protein.

**Supplementary Movie 5. Colocalization of TCRβ^+^ (FITC, green) and CTB^+^ (TRITC, orange) signals in the cytosol of sAb-stimulated T cells.** *Abbreviations*: CTB, cholera toxin B subunit; sAb, soluble anti-CD3/CD28 antibodies.

**Supplementary Movie 6. Colocalization of TCRβ^+^ (FITC, green) and CTB^+^ (TRITC, orange) signals in the released particles or inside the cytosol of iAb-stimulated CD4^+^ T cells.** *Abbreviations*: CTB, cholera toxin B subunit; iAb, immobile anti-CD3/CD28 antibodies.

**Supplementary Movie 7. Release of V5G^+^ microvilli particles from OTII CD4^+^ T cells during migration on the lipid bilayers presenting OVA_323–339_/I-A^b^/ICAM-1.** *Abbreviations*: ICAM, intercellular adhesion molecule; V5G, Vstm5 fused with green fluorescent protein.
